# Knowledge Distillation for Molecular Property Prediction: A Scalability Analysis

**DOI:** 10.1002/advs.202503271

**Published:** 2025-04-09

**Authors:** Rahul Sheshanarayana, Fengqi You

**Affiliations:** ^1^ College of Engineering Cornell University Ithaca NY 14853 USA; ^2^ Robert Frederick Smith School of Chemical and Biomolecular Engineering Cornell University Ithaca NY 14853 USA; ^3^ Cornell University AI for Science Institute Cornell University Ithaca NY 14853 USA; ^4^ Cornell AI for Sustainability Initiative (CAISI) Cornell University Ithaca NY 14853 USA

**Keywords:** graph neural networks, knowledge distillation, materials informatics, scalability

## Abstract

Knowledge distillation (KD) is a powerful model compression technique that transfers knowledge from complex teacher models to compact student models, reducing computational costs while preserving predictive accuracy. This study investigated KD's efficacy in molecular property prediction across domain‐specific and cross‐domain tasks, leveraging state‐of‐the‐art graph neural networks (SchNet, DimeNet++, and TensorNet). In the domain‐specific setting, KD improved regression performance across diverse quantum mechanical properties in the QM9 dataset, with DimeNet++ student models achieving up to an 90% improvement in $R^{2}$ compared to non‐KD baselines. Notably, in certain cases, smaller student models achieved comparable or even superior $R^{2}$ improvements while being 2× smaller, highlighting KD's ability to enhance efficiency without sacrificing predictive performance. Cross‐domain evaluations further demonstrated KD's adaptability, where embeddings from QM9‐trained teacher models enhanced predictions for ESOL (log*S*) and FreeSolv (Δ*G_hyd_*), with SchNet exhibiting the highest gains of ≈65% in log*S* predictions. Embedding analysis revealed substantial student‐teacher alignment gains, with the relative shift in cosine similarity distribution peaks reaching up to 1.0 across student models. These findings highlighted KD as a robust strategy for enhancing molecular representation learning, with implications for cheminformatics, materials science, and drug discovery.

## Graphical abstract

This study explores the effectiveness of knowledge distillation (KD) for molecular property prediction using graph neural networks. By leveraging KD, student models achieve improved scalability and predictive accuracy across domain‐specific and cross‐domain molecular datasets. Embedding analysis highlights the enhanced alignment between teacher and student models, demonstrating KD's potential for efficient molecular representation learning.

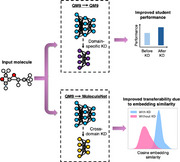

## 1 Introduction

Molecular graphs are pivotal in cheminformatics, representing molecules as nodes (atoms) and edges (bonds), enabling advanced machine learning models to predict molecular properties efficiently. Datasets such as QM9^[^ ^1^ ^]^ and MoleculeNet^[^ ^2^ ^]^ provide a rich variety of molecular structures annotated with diverse chemical and physical properties, facilitating both predictive model development and performance evaluation. Leveraging these datasets, state‐of‐the‐art models like SchNet,^[^ ^3^, ^4^ ^]^ DimeNet++,^[^ ^5^, ^6^ ^]^ and TensorNet^[^ ^7^ ^]^ have demonstrated significant success in molecular property prediction. SchNet captures continuous atomic interactions using convolutional layers, DimeNet++ integrates angular information to enhance geometric understanding, and TensorNet employs tensorial representations to capture higher‐order interactions, offering deeper structural insights.

However, the increasing complexity of molecular models presents significant computational challenges,^[^ ^8^, ^9^, ^10^, ^11^ ^]^ particularly for large‐scale datasets such as QM9 and MoleculeNet. These datasets contain extensive quantum mechanical and experimental property annotations across thousands of molecular structures, requiring models to process high‐dimensional representations that encompass atomic positions, bond interactions, and electronic properties. As a result, training and deploying machine learning models on such datasets demands substantial computational resources, including high memory usage, prolonged training times, and intensive GPU processing.^[^ ^12^ ^]^ Graph neural networks (GNNs) such as SchNet, DimeNet++, and TensorNet further intensify these challenges by incorporating intricate spatial and electronic interactions within molecular graphs. While these architectures provide state‐of‐the‐art performance in molecular property prediction, their computational demands hinder their scalability and practical application, particularly in high‐throughput screening pipelines and resource‐constrained environments.

To address these bottlenecks, model compression techniques such as pruning,^[^ ^13^, ^14^ ^]^ transfer learning,^[^ ^15^, ^16^, ^17^, ^18^ ^]^ and knowledge distillation (KD)^[^ ^19^, ^20^, ^21^, ^22^, ^23^, ^24^, ^25^, ^26^, ^27^, ^28^, ^29^, ^30^, ^31^, ^32^ ^]^ have gained prominence. However, pruning can lead to the loss of structural information by removing essential connections, resulting in irregular sparsity that limits hardware efficiency.^[^ ^33^ ^]^ Transfer learning, on the other hand, faces challenges such as negative transfer, where pre‐trained knowledge can hinder performance on new tasks.^[^ ^33^ ^]^ Unlike these approaches, KD preserves the teacher model's structured knowledge, facilitates smooth adaptation across datasets, and produces dense yet compact models optimized for computational efficiency and generalization. This approach is particularly advantageous for molecular datasets, where efficient prediction of quantum and physicochemical properties is crucial for accelerating material discovery and drug design.^[^ ^22^, ^23^, ^24^, ^25^, ^29^ ^]^ Beyond computational efficiency, KD also plays a crucial role in improving model generalizability,^[^ ^25^, ^31^ ^]^ interpretability,^[^ ^27^ ^]^ and robustness.^[^ ^30^ ^]^ In molecular property regression, different datasets often exhibit variations in numerical scales, feature importance, and noise levels, making direct model generalization challenging.^[^ ^1^, ^2^, ^31^ ^]^ By aligning student model embeddings with those of the teacher, KD facilitates smoother adaptation across property distributions, ensuring that student models retain essential predictive patterns while learning from diverse molecular representations. This property‐specific knowledge transfer is particularly valuable when transitioning from quantum mechanical datasets, such as QM9, to experimental datasets like ESOL and FreeSolv, where the distributional shift in molecular properties requires careful embedding refinement for accurate predictions.

Several studies have demonstrated the utility of KD in molecular modeling applications. The FusionDTA framework^[^ ^27^ ^]^ exemplifies its potential by integrating attention mechanisms to enhance drug‐target binding affinity predictions while maintaining computational efficiency. Similarly, MolKD^[^ ^25^ ^]^ applies KD to chemical reaction contexts, optimizing molecular property predictions by leveraging pre‐trained knowledge from reaction‐based data, reducing the need for extensive retraining on datasets such as QM9 and MoleculeNet. This property‐specific knowledge transfer is particularly valuable when transitioning from quantum mechanical datasets, such as QM9, to experimental datasets like ESOL and FreeSolv, where the distributional shift in molecular properties requires careful embedding refinement for accurate predictions. These studies underscore the potential of KD to enhance predictive performance while addressing the challenges of dataset heterogeneity in molecular property regression.

Beyond cheminformatics, KD has also demonstrated potential in materials science applications, particularly in molecular property regression, where high‐dimensional continuous outputs make efficient knowledge transfer challenging.^[^ ^23^, ^29^, ^30^, ^31^, ^34^ ^]^ By distilling complex models into smaller, more efficient ones, KD enables the retention of predictive accuracy while significantly reducing computational costs—an essential advantage in molecular modeling. Despite these advancements, the application of KD in materials informatics remains relatively unexplored, particularly for regression tasks involving electronic structure property predictions, where capturing complex quantum mechanical relationships poses a unique challenge. Expanding KD to such domains could enhance model efficiency while preserving high‐accuracy predictions, facilitating broader adoption in computational materials discovery. This efficiency has been demonstrated in materials science applications, such as Zhang and Saniie's work,^[^ ^30^ ^]^ where KD was applied to characterize steel microstructures using ultrasonic evaluation, leading to substantial reductions in data requirements and computational overhead. Similarly, Taniguchi^[^ ^29^ ^]^ applied KD to neural network potentials for organic molecular crystals, maintaining high accuracy while optimizing computational efficiency. In molecular generation, Wang et al.^[^ ^31^ ^]^ utilized KD in conditional transformers to enhance structure generation under constraints, and Yu and Börjesson^[^ ^23^ ^]^ explored KD for chemical transformer compression, accelerating high‐throughput virtual screening.

Despite these advancements, applying KD to regression tasks in cheminformatics and materials science presents unique challenges.^[^ ^22^, ^23^, ^24^, ^29^ ^]^ Unlike classification tasks, where loss functions such as cross‐entropy are well‐established, regression requires tailored loss functions to ensure the accurate transfer of continuous‐valued numerical relationships.^[^ ^35^, ^36^ ^]^ Additionally, balancing model compression with predictive accuracy is critical, especially in high‐dimensional molecular property spaces.^[^ ^23^, ^31^ ^]^ Zhang and Saniie^[^ ^30^ ^]^ further emphasize the need for adaptable KD frameworks to accommodate diverse materials and properties without extensive customization.

This study investigates KD's ability to enhance regression‐based molecular property prediction across different dataset distributions, addressing key challenges such as embedding transferability, and architecture‐dependent KD effectiveness. Using teacher models trained on selected QM9 properties, we evaluate the performance of student models across additional QM9 features (domain‐specific KD) and extend the study to experimental datasets in MoleculeNet such as ESOL and FreeSolv (cross‐domain KD). This analysis aligns with broader research in domain adaptation in cheminformatics, where cross‐domain KD has the potential to bridge theoretical and experimental molecular representations, ensuring more generalizable and robust models.^[^ ^18^, ^37^ ^]^ Overall, our work provides a comprehensive analysis of KD's effectiveness in optimizing embeddings and enhancing generalizability across diverse molecular domains.

Building on the challenges identified in previous studies, our work introduces a versatile framework for applying KD in predicting a range of properties derived from quantum mechanics and physical chemistry, leading to the following key findings:

1. **Enhanced regression performance with KD**: KD significantly improves regression‐based molecular property prediction, with student models achieving up to a 70% relative improvement in *R* ^2^ for properties like aqueous solubility (log*S*) in cross‐domain settings, showcasing KD's efficacy to generalize across complex continuous outputs.
2. **Architecture‐dependent effectiveness of KD**: Smaller DimeNet++ models exhibit higher performance gains for simpler properties like internal energy (*U*/*U*_0_), likely due to their inherent inductive biases. In contrast, larger SchNet models demonstrate superior embedding alignment—where the student model effectively captures the teacher's feature space—and achieve greater performance improvements for more complex properties (*U^ATOM^*/*H^ATOM^*). This highlights the architecture‐specific impact of KD, suggesting that different models benefit from KD in distinct ways depending on the nature of the target property.
3. **Cross‐domain transfer of embeddings**: Embeddings trained on theoretical quantum properties (QM9) effectively transfer to experimental datasets (ESOL and FreeSolv), achieving substantial *R* ^2^ improvements, particularly for log*S*, demonstrating KD's adaptability to distinct property distributions and domains.
4. **Improved embedding alignment due to KD**: Cosine similarity analysis reveals consistent improvements in embedding alignment between teacher and student models, with larger student models benefiting more in terms of embedding refinement, especially in cross‐domain scenarios where property distributions differ significantly.

## 2 Results and Discussion

In this section, we first present the knowledge distillation (KD) framework applied to domain‐specific and cross‐domain molecular property prediction. We then evaluate KD's effectiveness for domain‐specific regression tasks by training teacher models on QM9 properties and distilling embeddings into student models for predicting additional QM9 properties. Focusing on ten quantum mechanical properties (Table 2), we assess how KD guides student models across three GNN architectures. For cross‐domain evaluation, we transfer QM9 embeddings to ESOL and FreeSolv to test KD's adaptability. Our analysis highlights KD's impact on regression accuracy, embedding alignment, and generalizability across molecular datasets.

### 2.1 Model Architectures and Knowledge Distillation Framework

This study employs three state‐of‐the‐art GNN architectures—SchNet, DimeNet++, and TensorNet—as the foundation for our KD framework. Each architecture was selected for its unique capabilities in molecular property prediction, enabling a comprehensive evaluation of KD across both domain‐specific and cross‐domain tasks.

SchNet^[^ ^3^, ^4^ ^]^ is a continuous‐filter convolutional network designed specifically for quantum chemistry tasks. It dynamically learns interatomic interactions using atomic positions and features, making it highly effective for predicting spatially dependent quantum mechanical properties. As a simpler and computationally efficient architecture, SchNet is well‐suited for tasks requiring lightweight student models. In contrast, DimeNet++^[^ ^5^, ^6^ ^]^ incorporates angular information alongside interatomic distances, enabling it to capture intricate spatial relationships and electronic interactions. Its directional message‐passing mechanism makes it particularly adept at handling geometry‐dependent properties, which is further amplified through KD. Lastly, TensorNet,^[^ ^7^ ^]^ a tensor‐based GNN, is optimized for handling high‐dimensional molecular embeddings. Its ability to capture higher‐order interactions between atoms and scalability across large datasets makes it an ideal candidate for complex molecular property prediction. **Table** **1** summarizes the number of parameters and training times for each architecture, highlighting the computational trade‐offs between model complexity and efficiency, both with and without KD.

**Table 1:** Summary of model architecture, both student and teacher, along with the corresponding number of trainable parameters in case of domain‐specific (QM9) and cross‐domain (ESOL/FreeSolv) applications.

Architecture	Model type	Model name	Model Parameters		GPU training time (s)			
					QM9		ESOL/FreeSolv	
			QM9	ESOL/FreeSolv	Without KD	With KD	Without KD	With KD
SchNet	Teacher	‐	456069		1128.0			
	Student	Model 1a	136714	136129	732.0	990.0	6.0	7.2
		Model 1b	152650	152065	744.0	1008.0	6.0	7.8
		Model 1c	186826	186241	714.0	978.0	6.6	7.8
		Model 1d	264394	263809	726.0	996.0	7.8	8.4
DimeNet++	Teacher	‐	727158		3042.0			
	Student	Model 2a	237510	236934	1422.0	2280.0	82.2	222.0
		Model 2b	404326	403462	2046.0	3012.0	96.0	163.2
		Model 2c	571142	569990	2238.0	3150.0	115.2	174.0
TensorNet	Teacher	‐	1230725		5958.0			
	Student	Model 3a	528650	527489	2520.0	4692.0	42.0	70.2
		Model 3b	762890	761729	3882.0	5970.0	57.0	85.8
		Model 3c	997130	995969	5256.0	6762.0	75.0	100.8

To create computationally efficient student models while preserving key molecular representation capabilities, we systematically reduced model complexity in a way that maintained each architecture's fundamental strengths. For SchNet, we decreased the number of continuous filter basis functions from 128 in the teacher model to 8, 16, 32, and 64 in student models 1a, 1b, 1c, and 1d, respectively. These filters control the resolution at which atomic interactions are learned through radial basis expansions. We selected this reduction strategy because SchNet's representational power lies primarily in its continuous convolution filters, and adjusting the number of filters allowed us to reduce model complexity without altering the message‐passing mechanism. This setup enabled us to examine the trade‐off between interaction resolution and computational cost while assessing whether KD could preserve predictive performance under these constraints. For DimeNet++, we reduced the number of interaction blocks—responsible for directional message passing and capturing angular dependencies—from 4 in the teacher model to 1, 2, and 3 in student models 2a, 2b, and 2c. Since these blocks are the core mechanism for encoding geometric features, this reduction allowed us to explore how much spatial and angular information could be retained with shallower architectures, and how effectively KD can bridge the gap introduced by this reduction in geometric expressivity. For TensorNet, we similarly decreased the number of interaction blocks from 4 in the teacher model to 1, 2, and 3 in student models 3a, 3b, and 3c. These blocks perform iterative message passing to refine molecular representations through multi‐body interactions. In contrast to SchNet, TensorNet's capacity is governed more by the number of refinement layers, making interaction block reduction a natural strategy to compress the model while relying on KD to recover lost representational depth. Across all three architectures, these carefully selected reduction strategies ensured that the student models remained computationally lightweight while retaining the ability to capture complex molecular interactions. The number of parameters for each model is summarized in Table 1.

**Figure 1:**
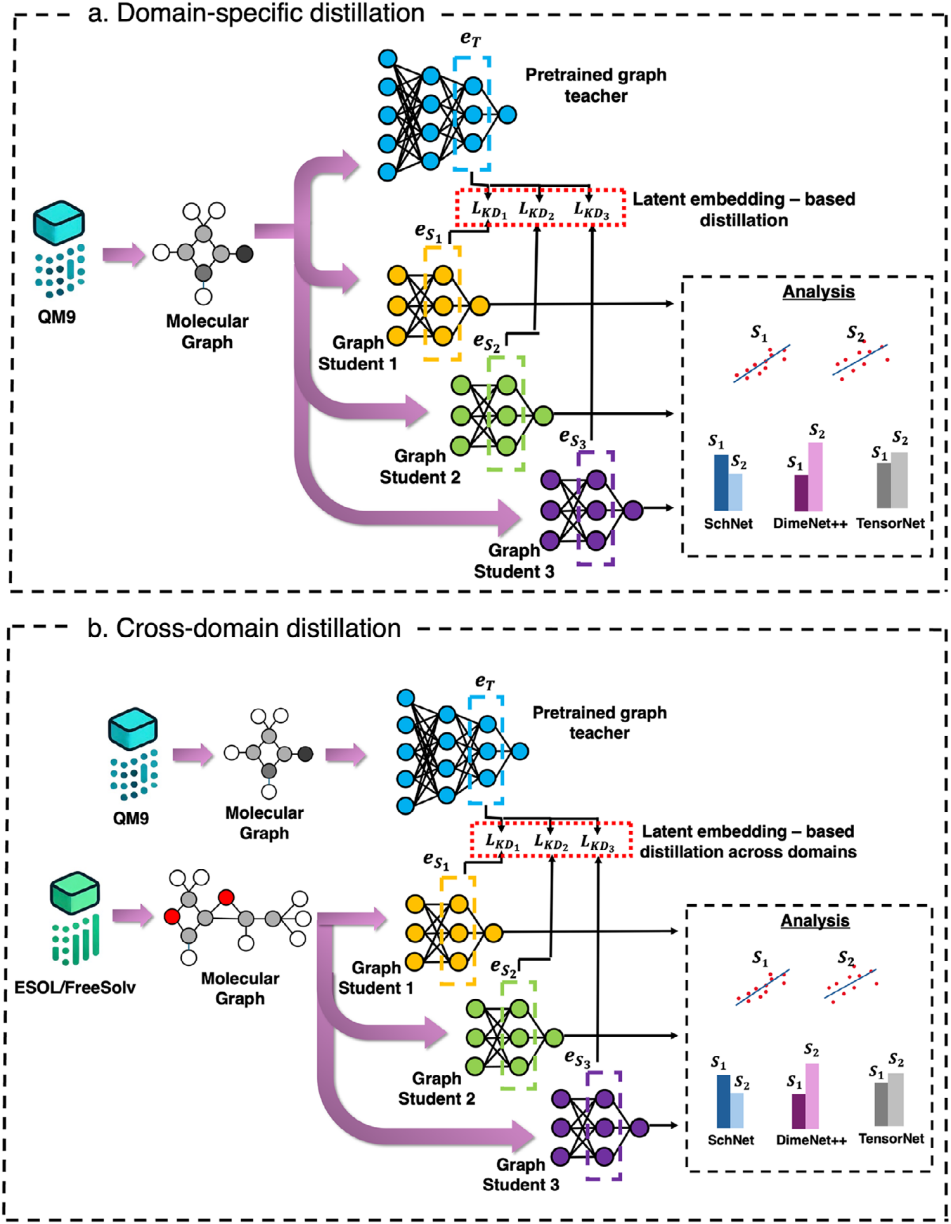
Overview of the KD framework architecture. a) Domain‐specific KD, where a pretrained graph‐based teacher model trained on QM9 properties transfers latent embeddings to multiple student models tasked with predicting additional QM9 properties. b) Cross‐domain KD, where embeddings from the QM9‐trained teacher model are transferred to student models for predicting molecular properties from external datasets, such as ESOL and FreeSolv. The latent embedding‐based distillation process aligns teacher (*e_r_*) and student representations ($e_{S_{i}}$ corresponding to the *i^th^* student model). The analysis block evaluates the similarity of student and teacher embeddings and performance across both setups.

The study employs two distinct setups of KD methodologies to evaluate its efficacy in molecular property prediction: domain‐specific distillation and cross‐domain distillation, as depicted in **Figure** **1**. In the domain‐specific distillation setup (Figure 1a), the teacher model, pretrained on five quantum mechanical properties from the QM9 dataset, transfers knowledge to three student models with progressively increasing complexity and capacity. The latent embeddings (*e_T_*) generated by the teacher model are distilled into the student models ($e_{S_{i}},\;i=1,\;2,\;\text{…}$) using a latent embedding‐based KD loss (*L_KD_*) shown in Equation (3), which measures the alignment between teacher and student embeddings. These embeddings serve as the foundation for improving the prediction of ten additional unseen QM9 properties, which are distinct from the properties used to train the teacher.

In the cross‐domain distillation setup (Figure 1b), the pretrained QM9 teacher model is leveraged to optimize student models trained on two external datasets—ESOL and FreeSolv—which contain molecular properties (log*S* and Δ*G_hyd_*, respectively). This setup tests the ability of KD to generalize embeddings across datasets with varying molecular characteristics and distributions. Like the domain‐specific approach, the latent embeddings generated by the QM9‐trained teacher model are distilled into the students. Here, the focus is on analyzing the adaptability of embeddings and the student models' performance in predicting properties from datasets with distinct chemical and physical properties. The effectiveness of KD is evaluated using *R* ^2^ metrics for property prediction and embedding alignment measures. Additionally, we assess the computational efficiency of different student models by measuring their training times across QM9 and cross‐domain datasets (Table 1). This provides insights into the scalability of KD across architectures and datasets with varying molecular complexities.

This dual methodology enables a systematic evaluation of KD's impact both within a consistent molecular space and across varying molecular domains, providing insights into its adaptability and robustness in improving regression‐based molecular property predictions.

### 2.2 Enhancing ***R*** ^2^ and Computational Efficiency with Domain‐Specific Knowledge Distillation

In this section, we investigate the effectiveness of teacher models in guiding student models within the same molecular property space, focusing on ten quantum mechanical properties from the QM9 dataset mentioned in **Table** **2**. Additionally, we also explore how KD enhances regression‐based predictions for molecular properties within the QM9 dataset across three distinct GNN architectures. The analysis assesses both the performance improvement across various molecular properties and the influence of model size on KD effectiveness, offering a comprehensive understanding of KD's role in molecular property prediction. To quantify the impact of KD, we calculate the relative $R^{2}$ deviation between student models with and without KD as shown in Equation (1) below. However, note that the magnitude of this deviation is inherently linked to the absolute *R* ^2^ values of the student models. As presented in Table S1 (Supporting Information), models with lower baseline *R* ^2^ values exhibit more pronounced relative deviations. In contrast, models with already high *R* ^2^, such as TensorNet trained on QM9, show smaller deviations after applying KD since the potential for further improvement is limited. Including absolute *R* ^2^ values provide a more detailed assessment of KD's effectiveness beyond relative gains.

(1) $$R^{2}\;\mathrm{deviation}\;\;\left(\%\right)=\left(1-\frac{R_{\mathrm{without}\;\mathrm{KD}}^{2}}{R_{\mathrm{with}\;\mathrm{KD}}^{2}}\right)\;\times 100$$

**Table 2:** Datasets and properties used for domain‐specific and cross‐domain KD. The QM9 dataset supports domain‐specific KD, with five molecular properties for teacher training and ten additional properties for evaluating KD on student models. Cross‐domain KD evaluates the transfer of QM9‐trained embeddings to predict molecule solubility (ESOL) and hydration free energy (FreeSolv). The units corresponding to each property is also specified.

Domain	Dataset	Number of molecules	Property	Unit
Domain‐specific	QM9 (teacher training)	130831	Dipole moment (μ)	D
			Isotropic polarizability (α)	Å^3^
			HOMO energy (ε_*HOMO*_)	eV
			LUMO energy (ε_*LUMO*_)	eV
			HOMO‐LUMO gap (Δε)	eV
Domain‐specific	QM9 (student training and evaluation)	130831	Electronic spatial extent (〈*R* ^2^〉)	Å^3^
			Internal energy at 0 K (*U*_0_)	eV
			Internal energy at 298.15 K (*U*)	eV
			Enthalpy at 298.15 K (*H*)	eV
			Free energy at 298.15 K (*G*)	eV
			Heat capacity at 298.15 K (*c_v_*)	kcal/mol · K
			Atomization energy at 0 K ($U_{0}^{ATOM}$)	eV
			Atomization energy at 298.15 K (*U^ATOM^*)	eV
			Atomization enthalpy at 298.15 K (*H^ATOM^*)	eV
			Atomization free energy at 298.15 K (*G^ATOM^*)	eV
Cross‐domain	ESOL (student training and evaluation)	1128	Aqueous solubility (log*S*)	log(mol L^−1^)
	FreeSolv (student training and evaluation)	642	Hydration free energy (Δ*G_hyd_*)	Kcal mol^−1^

**Figure 2:**
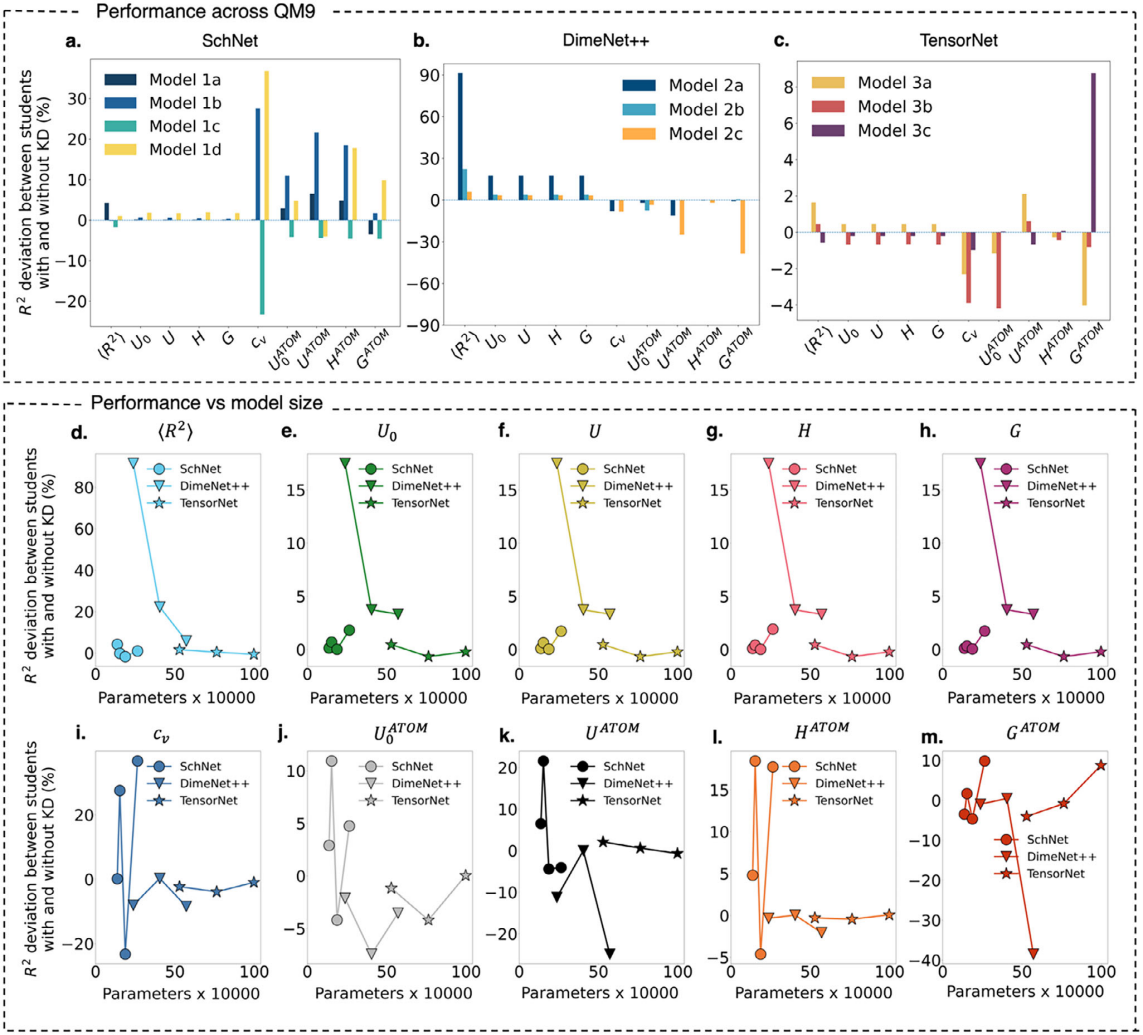
Analysis of KD performance across ten QM9 molecular properties and student model sizes for SchNet a), DimeNet++ b), and TensorNet c) architectures. Panels (a‐c) Percentage deviation in $\;R^{2}$ between student models trained with and without KD for each molecular property. Panels (d‐m) Breakdown of $R^{2}$ deviation trends across model sizes for each property, plotted separately to highlight individual behaviors. Different markers represent SchNet (circles), DimeNet++ (triangles), and TensorNet (stars), enabling a direct comparison of KD effectiveness across architectures.

For SchNet, KD yields consistent improvements across most molecular properties, as shown in **Figure** **2**a. Properties like heat capacity (*c_v_*) and atomization energy (*U^ATOM^*) benefit significantly, particularly for larger student models, which show 37% and 25% deviations in *R* ^2^, respectively. This suggests that KD for SchNet enhances the student models’ ability to capture complex molecular patterns, leveraging the embeddings distilled from the teacher model. Conversely, properties such as internal energy (*U*_0_/*U*) and enthalpy (*H*) exhibit near‐zero deviations, implying that these properties are already well‐represented in the student models, leaving minimal room for KD to provide further improvements.

DimeNet++ exhibits a distinct response to KD, as depicted in Figure 2b, with smaller student models outperforming their larger counterparts for simpler properties like internal energy (*U*/*U*_0_) and electronic spatial extent (〈*R* ^2^〉). Notably, the improvement in 〈*R* ^2^〉 is especially pronounced, reaching 90%. These results suggest that smaller models benefit more from teacher‐guided embeddings for such properties, achieving greater predictive accuracy enhancements. However, for complex properties like atomization energy (*U^ATOM^*) and enthalpy (*H^ATOM^*), the deviations are minimal or negligible, regardless of model size, reflecting the inherent ability of DimeNet++ to capture the necessary molecular interactions. These findings emphasize the effectiveness of KD in aiding smaller student models while highlighting the diminishing returns of KD as model size increases or for properties that are intrinsically well‐represented.

However, TensorNet's results, depicted in Figure 2c, demonstrate a stable response across molecular properties, with deviations remaining close to zero for most properties. This indicates that TensorNet's tensor‐based representation is robust, capturing key molecular features effectively without heavy reliance on KD. Unlike SchNet and DimeNet++, which rely on message passing and spatial encoding, TensorNet may already encode sufficient structural and electronic information, reducing the necessity for knowledge transfer. Additionally, its parameterization might be less redundant, allowing even smaller student models to retain much of the teacher's predictive power.

**Figure 3:**
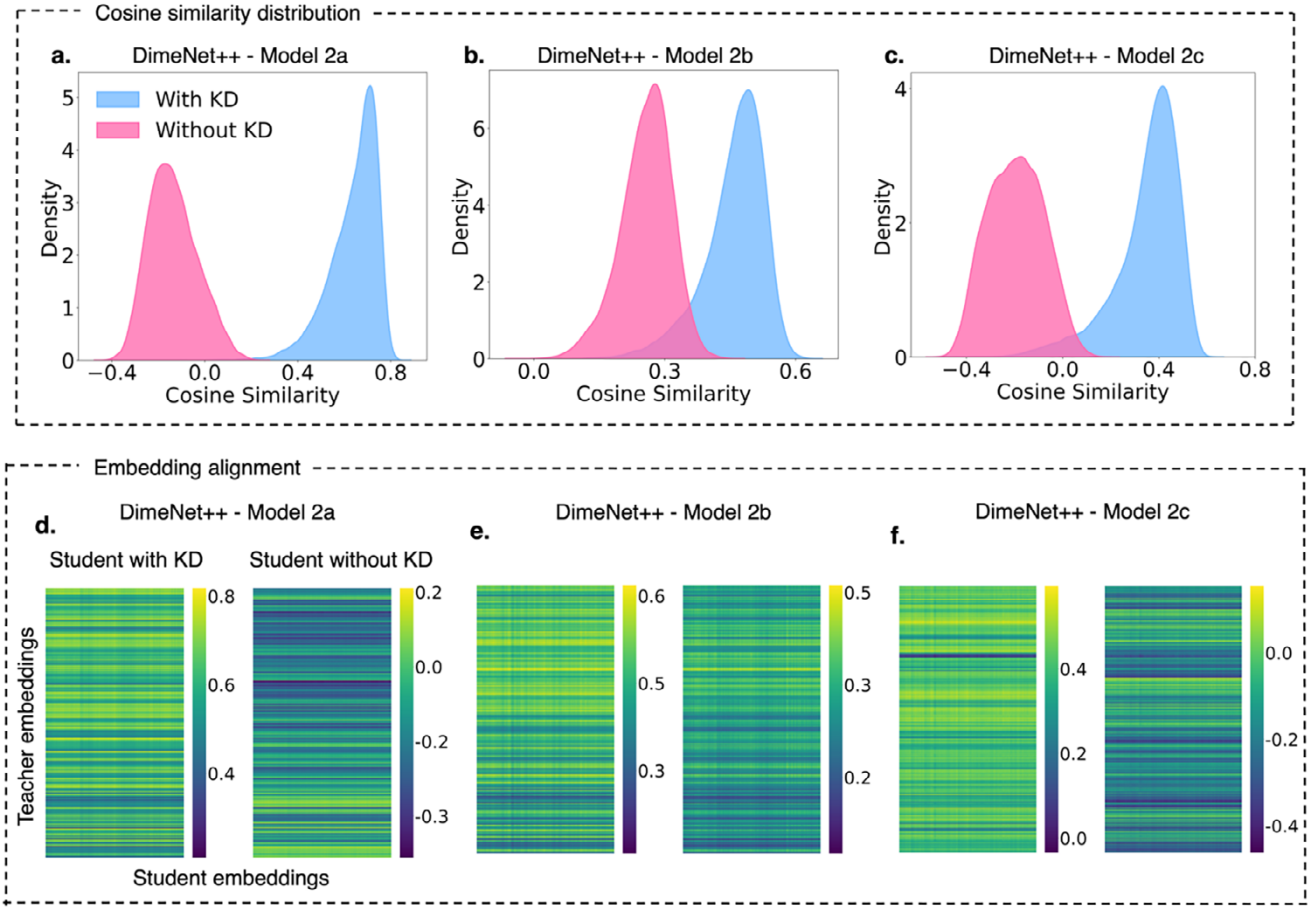
Cosine similarity analysis and embedding alignment across student and teacher embeddings for DimeNet++ models. Panels a–c) Cosine similarity distributions between teacher and student embeddings for three different DimeNet++ student models: Model 2a, Model 2b, and Model 2c. Panels (d–f) Heatmaps of pairwise cosine similarities between teacher and student embeddings for Models 2a, 2b, and 2c, respectively. Each subpanel compares the student trained using KD (left heatmap) and student trained without KD (right heatmap). Note that stronger embedding alignment suggests improved knowledge transfer, reducing training requirements for student models while maintaining predictive performance.

Building on the insights from Figure 2, where SchNet, DimeNet++, and TensorNet exhibited varying responses to KD, **Figure** **3** further explores the embedding space of DimeNet++. Among the three architectures, DimeNet++ displayed the most pronounced changes in scores with KD, making it an ideal candidate for a focused analysis of embedding alignment. This may be attributed to its explicit modeling of angular interactions, which enhances its sensitivity to knowledge transfer. Additionally, the architecture's higher parameter count could introduce redundancy, making it more receptive to KD by filtering out less relevant features while refining critical molecular representations. The figure examines cosine similarity between teacher and student embeddings across three DimeNet++ student models of increasing size: Models 2a, 2b, and 2c, highlighting how KD influences the structure of learned embeddings and enhances generalization.

In Figure 3a–c, the distributions of cosine similarity offer a clear narrative of KD's impact. For each student model, the distributions of student models trained with KD (blue) consistently shift toward higher cosine similarity values compared to those trained without KD (pink). This shift is rightward, and the distributions become wider as model size increases, with Model 2a showing the highest alignment to the teacher embeddings, with the cosine similarity peaks differing by 0.8. The trend underscores how KD facilitates more effective knowledge transfer in smaller models, where the reduced capacity enables the student to better replicate the teacher's latent space. Conversely, the distributions of models trained without KD remain slightly broader across all models, reflecting the limitations of unguided student training. These outcomes align with previous studies in deep learning, where embedding‐based KD has been shown to significantly improve model generalization in complex regression tasks.^[^ ^38^, ^39^ ^]^

Furthermore, the heatmaps in Figure 3d–f further substantiate these findings by visualizing pairwise cosine similarity between teacher and student embeddings. Each subpanel compares the corresponding results for models trained with KD (left heatmap) and without KD (right heatmap) for the three student models. For all model sizes, the heatmaps corresponding to models trained with KD exhibit higher average cosine similarity, indicating a more consistent alignment with the teacher embeddings. Notably, the improvement is most evident for Model 2a, where the embeddings with KD achieve the highest relative similarity and uniformity. On the other hand, the heatmaps of models trained without KD display significant variability and lower similarity, particularly for the smaller Model 2a and Model 2c, where the absence of teacher guidance hinders structured alignment.

Building on the detailed insights from Figures 2 and 3, this section demonstrates the transformative impact of KD on DimeNet++ embeddings, particularly through its ability to enhance alignment with teacher models across varying student sizes. In the next section, we extend this analysis to explore cross‐domain KD, where embeddings trained on quantum mechanical properties from QM9 are transferred to datasets with distinct experimental properties, such as ESOL (log*S*) and FreeSolv (Δ*G_hyd_*). This extension allows us to assess the adaptability and robustness of KD across molecular datasets with varying distributions and molecular characteristics.

### 2.3 Improving Generalization and Computational Efficiency with Cross‐Domain Knowledge Distillation

In this section, we evaluate the effectiveness of KD in adapting teacher embeddings trained on quantum mechanical properties from QM9 to predict properties in external datasets such as ESOL (log*S*) and FreeSolv (Δ*G_hyd_* ​). These datasets represent distinct molecular distributions and property scales, providing a robust test of KD's ability to generalize across domains. The analysis assesses how KD influences *R* ^2^ improvements (% deviations) in student models of varying sizes and architectures, revealing insights into its adaptability and performance in cross‐domain molecular property prediction.

**Figure 4:**
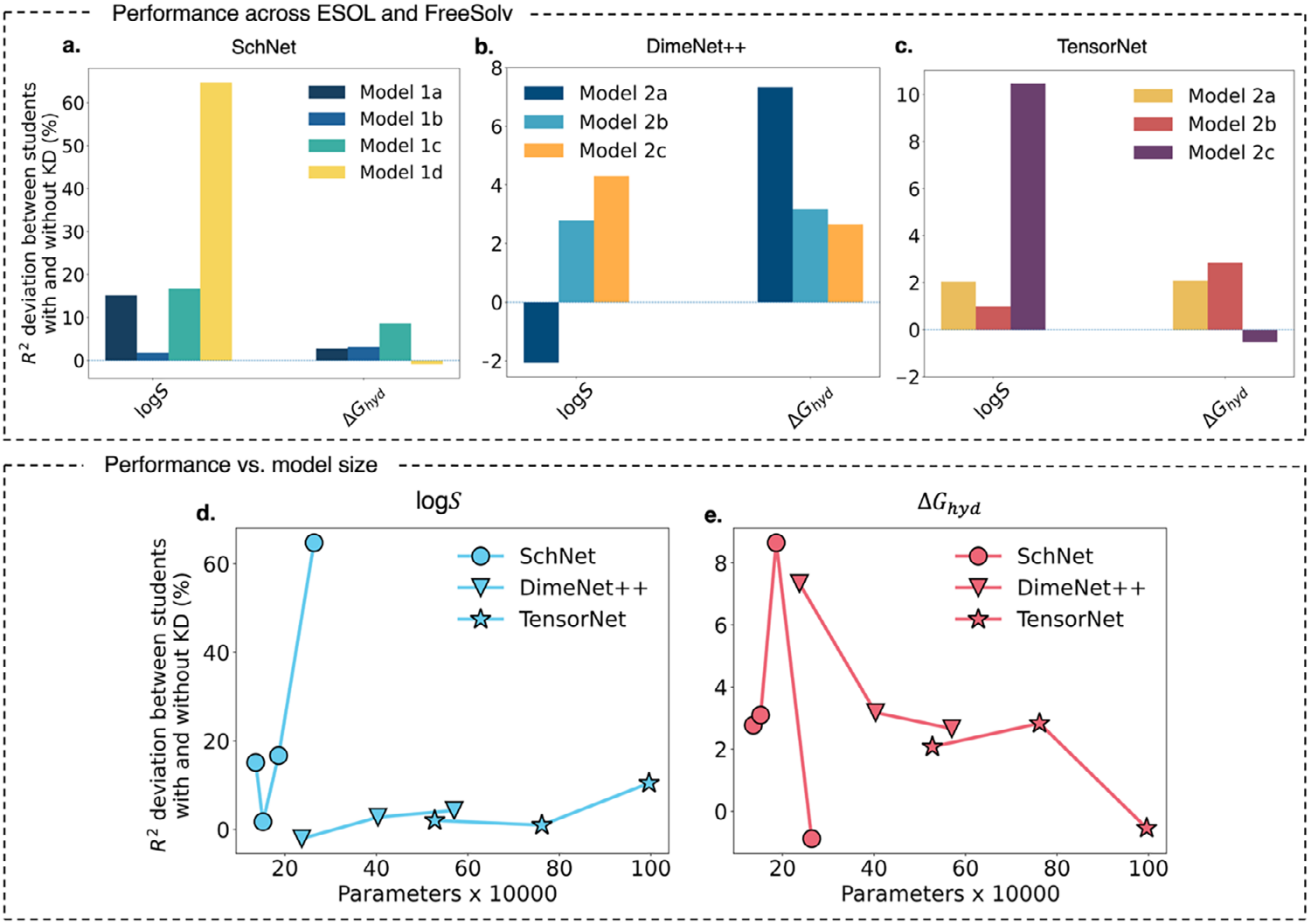
Analysis of KD performance across ESOL (log*S*) and FreeSolv (Δ*G_hyd_*) datasets and student model sizes for SchNet, DimeNet++, and TensorNet architectures. Panels a–c) Percentage deviation in *R* ^2^ between student models with and without KD for the two properties. Panels d,e) Relationship between the same *R* ^2^ deviation and the number of trainable parameters for each student model across the two cross‐domain properties – log*S* and Δ*G_hyd_*.

**Figure** **4** summarizes the cross‐domain results, showcasing the percentage deviation in $R^{2}$ scores between student models trained with and without KD across architectures (SchNet, DimeNet++, and TensorNet). For SchNet (Figure 4a), KD significantly improves predictions for ESOL (log*S*), with larger student models showing a pronounced increase in *R* ^2^ scores, peaking at Model 1d with an ≈65% improvement. This pronounced benefit for SchNet can be attributed to its continuous‐filter convolutional layers, which effectively capture atomic interactions but may initially struggle with learning molecular‐level solubility trends due to the absence of explicit global representations. The knowledge transfer from the teacher model likely helps mitigate this limitation by providing richer, more structured representations, enabling the student models to generalize better. In contrast, for FreeSolv (Δ*G_hyd_*), the impact of KD remains more subdued, as reflected in the smaller deviations across all student models. This could be due to Δ*G_hyd_* being more influenced by localized molecular interactions, which SchNet inherently models well even without KD. Furthermore Figure 4d,e reinforces these findings, showing a steep upward trend in $R^{2}$ improvements for log*S*, while Δ*G_hyd_* remains relatively flat, suggesting that KD primarily aids SchNet when the target property requires a broader contextual understanding rather than strictly local interactions.

In the case of DimeNet++ (Figure 4b), KD demonstrates a mixed response across both properties, with smaller student models (e.g., Model 2a) exhibiting slightly higher improvements for Δ*G_hyd_*, while larger models show greater gains for log*S*. This trend suggests that KD enables smaller models to better capture the nuanced interactions required for Δ*G_hyd_*, while the increased capacity of larger models allows them to leverage KD more effectively for log*S*, a property likely requiring more complex representational power. Furthermore, Figure 4d,e illustrates this complementary behavior, showing a steady increase in *R* ^2^ deviation for log*S* as model size grows, while *R* ^2^ deviations for Δ*G_hyd_* exhibit a decreasing trend. These observations highlight DimeNet++’s ability to adapt to varying property complexities under KD, emphasizing the architecture's versatility in cross‐domain molecular property prediction.

TensorNet (Figure 4c) exhibits a contrasting behavior, with KD yielding significant improvements for log*S* in the largest student model (Model 3c), reaching ≈10%. However, for Δ*G_hyd_*, KD has a negligible or even negative impact, as observed in the slight dips in *R* ^2^ deviations across student models. The line plots shown in Figure 4d,e highlights these divergent trends, where log*S* sees an upward trajectory, while Δ*G_hyd_* remains close to zero or slightly negative, suggesting that TensorNet's representation for this property may not benefit substantially from KD.

**Figure 5:**
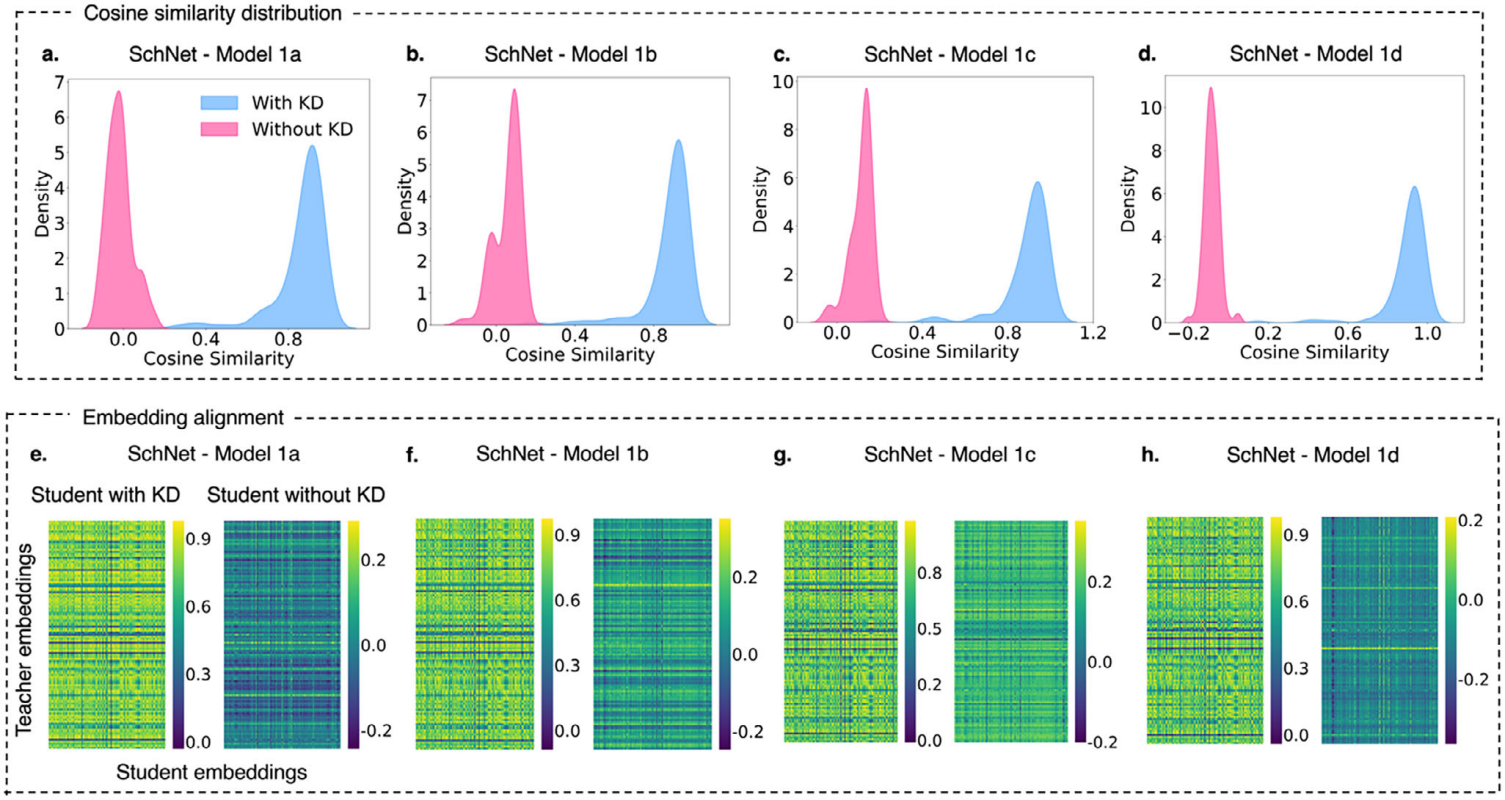
Cosine similarity distribution and embedding alignment for ESOL (log*S*) using SchNet student models. Panels a–d) Cosine similarity distributions between teacher and student embeddings with and without KD for increasing student model sizes (Model 1a to Model 1d). Panels e–h) Embedding alignment between teacher and student models for each student size, highlighting the differences in alignment with and without KD.

To further investigate the cross‐domain performance of KD, we analyze the embedding alignment between the QM9‐trained teacher and SchNet student models for the ESOL property (log*S*), as shown in **Figure** **5**. SchNet, the best‐performing architecture in this scenario, was selected to illustrate KD's role in refining student embeddings across different model sizes. This may stem from SchNet's continuous‐filter convolutional framework, which effectively captures spatial dependencies and benefits from KD‐driven embedding optimization. Additionally, the architecture's relatively lower parameter redundancy compared to DimeNet++ may contribute to its efficient adaptation to new molecular distributions. The figure presents two key perspectives: cosine similarity distributions (Figure 5a–d) and embedding alignment visualized as heatmaps (Figure 5e–h), highlighting how KD improves the structural consistency of student embeddings in cross‐domain settings.

The cosine similarity distributions, illustrated in Figure 5a–d, reveal a distinct improvement in alignment between SchNet teacher and student embeddings under KD. For the smallest student model, Model 1a (Figure 5a), KD results in a pronounced rightward shift of the distribution, signifying higher alignment with the teacher embeddings. In contrast, the distribution without KD is narrow and concentrated at lower similarity values, reflecting limited alignment. As the student model size increases (Figure 5b–d), the similarity distributions with KD continue to exhibit higher values compared to their no‐KD counterparts. Notably, the largest student model, Model 1d (Figure 5d), shows the most substantial benefits from KD, with a more pronounced relative cosine similarity shift of 1.0 toward higher similarity values compared to the smaller models. This aligns with the observed *R* ^2^ results, where larger SchNet models demonstrated more significant performance improvements under KD, indicating their ability to leverage the additional teacher‐guided knowledge more effectively.

The embedding alignment heatmaps in the bottom row of Figure 5 further corroborates these findings. For the smallest student model, Model 1a (Figure 5e), the KD heatmap displays bright and consistent diagonal patterns, indicative of strong correspondence between the teacher and student embeddings. In contrast, the no‐KD heatmap shows weaker alignment, with scattered and less intense patterns. As the student models increase in size (Figure 5f–h), the alignment improves significantly, with the largest model, Model 1d (Figure 5h), exhibiting the strongest alignment under KD. This suggests that larger models benefit more substantially from KD, both in terms of embedding alignment and performance improvements, due to their enhanced capacity to absorb and utilize teacher‐guided embeddings.

**Figure 6:**
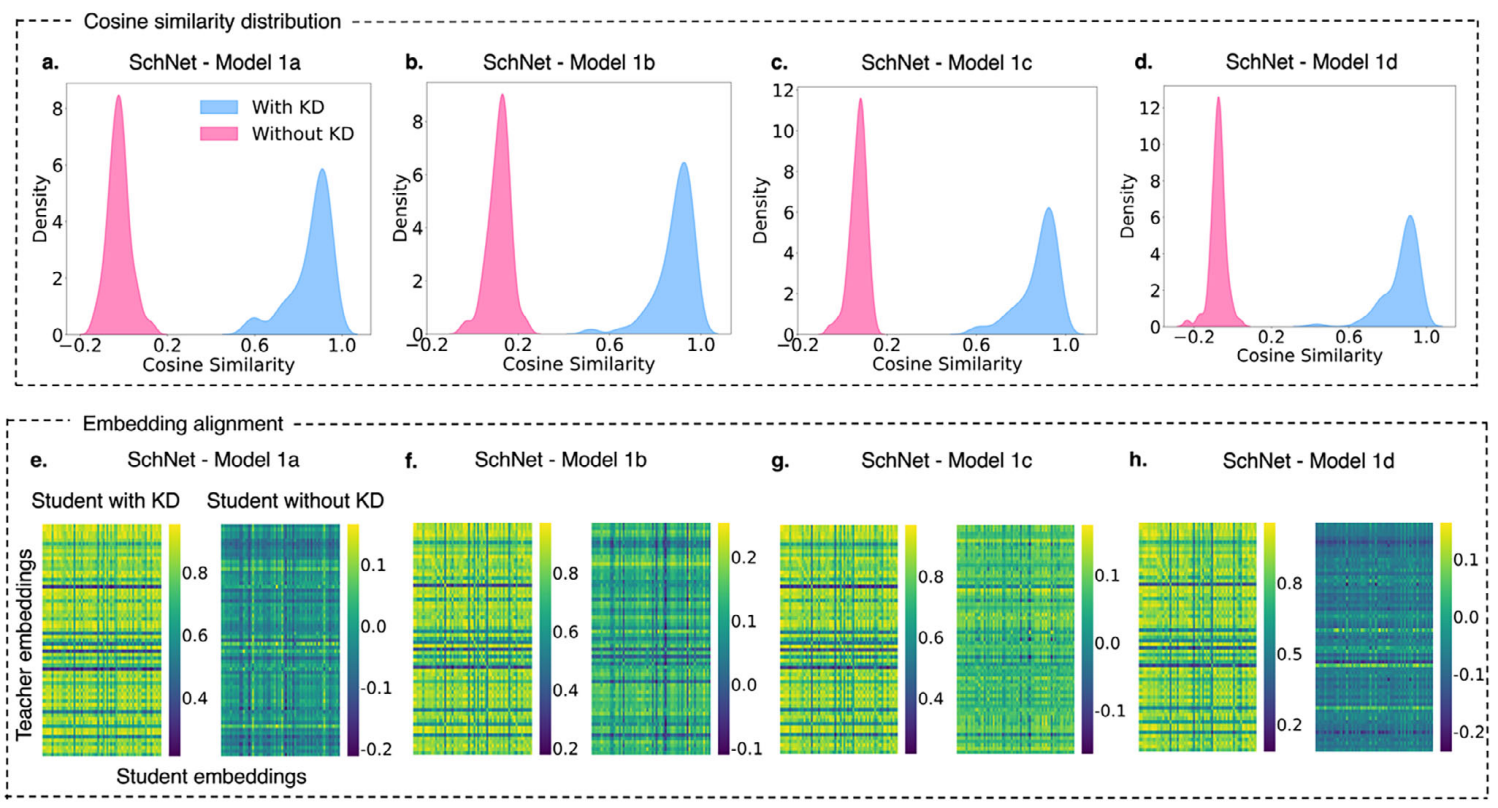
Cosine similarity distribution and embedding alignment for FreeSolv (Δ*G_hyd_*) using SchNet student models. Panels a–d) Cosine similarity distributions between teacher and student embeddings with and without KD for increasing student model sizes (Model 1a to Model 1d). Panels e–h) Embedding alignment between teacher and student models for each student size, highlighting the differences in alignment with and without KD.

To evaluate the impact of KD on FreeSolv (Δ*G_hyd_*) performance, we analyze the embedding alignment for SchNet student models of varying sizes, as depicted in **Figure** **6**. This analysis sheds light on how KD influences both embedding quality and downstream performance in cross‐domain tasks. Interestingly, this case contrasts with log*S* (see Figure 5), as the benefits of KD vary based on student model size.

The cosine similarity distributions in Figure 6a‐d reveal that KD consistently improves embedding alignment across all SchNet student models. For smaller models, such as Model 1a (Figure 6a), the cosine similarity distribution with KD shows a significant rightward shift compared to the no‐KD case, indicating enhanced alignment with teacher embeddings. However, as the model size increases (Figure 6b–d), this trend becomes more nuanced. Larger models, such as Model 1d (Figure 6d), demonstrate a much broader and higher similarity distribution under KD, reflecting stronger alignment compared to smaller models. Notably, the largest model exhibits a relative peak shift of 1.0 in cosine similarity, indicating a substantial improvement in embedding alignment. This observation highlights the greater capacity of larger models to leverage KD for embedding refinement, even in cross‐domain scenarios.

The embedding alignment heatmaps in Figure 6e–h further illustrate these findings. For the smallest model, Model 1a (Figure 6e), the KD heatmap reveals moderate alignment with the teacher embeddings, whereas the no‐KD heatmap shows minimal correspondence. As the models increase in size (Figure 6f–h), the diagonal patterns in the KD heatmaps become progressively more pronounced, indicating stronger alignment. The largest model, Model 1d (Figure 6h), achieves the highest alignment under KD, with highly consistent diagonal patterns. In contrast, the no‐KD heatmaps for all models exhibit weaker alignment, underscoring the role of KD in facilitating effective cross‐domain embedding transfer.

Despite these improvements in embedding alignment, the *R* ^2^ performance results for Δ*G_hyd_* reveal a subtle difference. Smaller models, such as Model 2a and Model 3a, demonstrate slightly better *R* ^2^ deviations under KD compared to larger models, as seen in Figure 4b,c, respectively. This suggests that while larger models excel in embedding alignment, smaller models may leverage KD more effectively for this specific property in terms of downstream prediction performance. This duality underscores the importance of balancing model size and KD efficacy to optimize both embedding alignment and property prediction in cross‐domain tasks.

Given the molecular diversity between computationally generated and experimentally measured datasets, a key question is whether KD primarily enhances predictions for QM9‐like molecules or if it facilitates generalization beyond the teacher's training distribution. The QM9 dataset consists of small organic molecules containing only carbon (C), hydrogen (H), oxygen (O), nitrogen (N), and fluorine (F), whereas the ESOL dataset includes a broader range of compounds with additional elements like sulfur (S), phosphorus (P), chlorine (Cl), and larger molecular structures. To investigate the extent of KD's impact across this domain shift, we divided the ESOL dataset into QM9‐like and non‐QM9‐like molecules based on molecular size and elemental composition. As shown in Figure S1 (see Supporting Information), KD generally leads to RMSE improvements across most student models in both molecular subsets, though a few exceptions are observed, particularly among larger models on QM9‐like molecules. On average, we observe RMSE reductions of 10–20% for QM9‐like molecules and up to 25% for non‐QM9‐like molecules, particularly in smaller student models. These improvements confirm that the learned representations from QM9 transfer effectively beyond its compositional space. Interestingly, the performance gain is more pronounced for non‐QM9‐like molecules, suggesting that distilled representations help student models better generalize to structurally diverse compounds outside the teacher model's training space. This highlights KD's ability to bridge the domain gap between computational and experimental datasets, especially in scenarios involving elements or molecular motifs absent in the teacher's training distribution.

While KD enhances predictive accuracy and embedding alignment across different architectures, its effectiveness can be influenced by several factors. One potential source of error could be overfitting in teacher models, where the teacher may learn spurious correlations or dataset‐specific biases that do not generalize well to student models. This is particularly relevant in complex molecular datasets like QM9, where variations in molecular interactions may not always be effectively transferred. Additionally, loss function misalignment presents another challenge—common KD approaches often optimize embedding similarity, but the underlying regression task requires precise numerical predictions. The balance between regression loss and distillation loss can significantly affect the extent to which student models benefit from KD. Addressing these issues may require adaptive distillation strategies, such as dynamically weighting the KD loss based on property‐specific characteristics or integrating uncertainty‐aware embedding transfers to mitigate overfitting effects.

## 3 Conclusion

This study demonstrates that KD effectively enhances molecular property prediction by improving scalability, reducing computational costs, and enabling cross‐domain generalization. By leveraging state‐of‐the‐art GNN architectures—SchNet, DimeNet++, and TensorNet—our framework addresses key challenges in regression‐based KD, including the effective transfer of fine‐grained numerical relationships and embedding optimization for continuous‐valued outputs.

In the domain‐specific setting, KD consistently improved performance across diverse quantum mechanical properties of the QM9 dataset. The results demonstrated that larger student models, particularly in SchNet, benefited significantly from teacher‐guided embeddings for complex molecular properties, while smaller DimeNet++ models excelled in predicting simpler properties. The analysis of relative *R* ^2^ deviations and embedding alignment further emphasized the nuanced impact of KD, with architectural differences influencing the extent of improvements. In the cross‐domain context, the robustness of KD was validated by transferring embeddings from QM9‐trained teacher models to predict experimental properties such as aqueous solubility (log*S*) and hydration free energy (Δ*G_hyd_*) from ESOL and FreeSolv datasets, respectively. SchNet emerged as the most adaptable architecture, exhibiting significant performance enhancements and improved embedding alignment, particularly for log*S*. DimeNet++ and TensorNet also demonstrated their versatility, with notable trends in embedding refinement and property‐specific improvements under KD.

Beyond these immediate findings, an important consideration is the scalability of this framework to larger datasets and more complex architectures. Additionally, KD could play a crucial role in scenarios where labeled data are scarce, such as drug discovery applications with limited experimental measurements for novel targets.^[^ ^40^, ^41^ ^]^ Unlike conventional deep learning approaches that require extensive labeled datasets, KD allows smaller student models to inherit meaningful representations from well‐trained teacher models, mitigating the limitations of sparse data. This becomes particularly relevant in few‐shot learning settings,^[^ ^42^ ^]^ where training a deep model from scratch is infeasible due to data constraints. While QM9 and MoleculeNet serve as a valuable benchmarks, real‐world molecular modeling often requires learning from extensive datasets such as PubChem^[^ ^43^ ^]^ and ChEMBL.^[^ ^44^ ^]^ KD has the potential to facilitate training on such large‐scale molecular libraries by enabling smaller, more efficient student models without compromising predictive accuracy. Similarly, with the growing adoption of graph transformers in molecular representation learning,^[^ ^45^, ^46^ ^]^ the integration of KD into transformer‐based architectures could further enhance computational efficiency. Unlike message‐passing GNNs, transformers rely on global attention mechanisms,^[^ ^47^ ^]^ which may respond differently to knowledge transfer. Future studies should explore whether KD can improve their learning efficiency, particularly in capturing long‐range dependencies.

Computational efficiency remains a fundamental concern when scaling KD‐based models. While large teacher models can be efficiently trained with modern hardware, real‐world deployment often demands lightweight models capable of rapid inference at scale.^[^ ^48^ ^]^ In applications such as high‐throughput virtual screening,^[^ ^49^ ^]^ real‐time molecular docking,^[^ ^50^ ^]^ or embedded AI in lab‐on‐a‐chip devices,^[^ ^51^ ^]^ distilling knowledge into compact student models—without substantial loss in accuracy—positions KD as a vital tool for balancing computational efficiency and predictive performance. Although this study highlights KD's ability to enhance student performance while reducing parameter complexity, its impact on training time and memory consumption requires further evaluation for larger‐scale implementations. Strategies such as multi‐teacher distillation,^[^ ^52^ ^]^ hierarchical KD,^[^ ^53^, ^54^ ^]^ and hybrid compression techniques^[^ ^55^ ^]^ could further optimize performance trade‐offs as models and datasets expand. Additionally, addressing regression‐specific challenges in KD—such as preserving fine‐grained numerical trends, refining loss functions to balance local and global errors,^[^ ^35^ ^]^ and improving the transferability of uncertainty‐aware embeddings—will be crucial for extending KD to high‐precision molecular modeling tasks.

Another promising direction involves integrating graph‐based attention mechanisms into KD pipelines. By leveraging attention‐based knowledge transfer, future studies could explore how self‐attention within GNNs^[^ ^46^, ^56^ ^]^ or hybrid Transformer‐GNN architectures^[^ ^23^, ^45^ ^]^ can further enhance the distillation process. This could be particularly impactful for tasks requiring multi‐scale molecular representations, such as protein‐ligand binding affinity prediction and reaction modeling, where hierarchical feature aggregation plays a critical role.

Overall, this work underscores the scalability and efficiency of KD in molecular property prediction, bridging the gap between computational complexity and predictive accuracy. By optimizing embeddings for diverse datasets, KD enhances model adaptability and paves the way for broader applications in cheminformatics, materials science, and related fields. Future work could focus on extending this framework to other molecular tasks, increasing model complexity through embedding size, integrating additional loss functions tailored for regression, and systematically investigating the interplay between model architecture,^[^ ^57^ ^]^ dataset complexity, and KD efficacy.

## 4 Experimental Section

### Datasets for Model Training and Evaluation

This study utilizes a combination of datasets to evaluate the performance and generalizability of the proposed KD framework. The datasets include quantum mechanical properties from QM9^[^ ^1^ ^]^ and molecular property datasets from MoleculeNet,^[^ ^2^ ^]^ such as ESOL and FreeSolv, alongside additional QM9 features not used in training the teacher model. The integration of these datasets allows for a comprehensive assessment of KD across diverse regression tasks, encompassing quantum, and solubility predictions.

The QM9 dataset serves as the foundation for this study, offering quantum mechanical properties of small organic molecules to evaluate the efficacy of KD in optimizing embeddings for regression tasks. It provides a chemically diverse set of over 130000 equilibrium molecular structures, including constitutional isomers of C, H, O, N, and F atoms, making it a comprehensive benchmark for molecular modeling. The dataset spans a wide range of quantum mechanical properties, including electronic, energetic, and thermodynamic descriptors, ensuring a broad numerical spectrum that facilitates knowledge transfer across different molecular property distributions. The teacher model is trained on five selected quantum properties, covering a wide spectrum of numerical ranges and physical significance, as shown in Table 2. These properties provide a robust foundation for embedding optimization and serve as a generalizable knowledge base for distillation into student models, allowing for improved predictive performance on both seen and unseen molecular properties.

To assess the domain‐specific performance of KD on the student model, ten additional QM9 properties, not included during teacher training, were selected for evaluation, also detailed in Table 2. This setup ensures a comprehensive evaluation of KD within the QM9 domain, examining the generalization capabilities of the student model across unseen quantum properties.

For cross‐domain KD, embeddings from the QM9‐trained teacher model were transferred to predict distinct chemical and physical properties from external datasets, specifically ESOL and FreeSolv from the MoleculeNet benchmark. These datasets were selected as they represent experimentally derived properties—aqueous solubility (log*S*) and hydration free energy (Δ*G_hyd_*)—which contrast with the theoretical quantum properties of QM9. Their relevance to real‐world applications, such as drug formulation and materials design, makes them ideal for assessing KD's adaptability beyond theoretical simulations. Additionally, ESOL and FreeSolv pose unique cheminformatics challenges due to experimental variability providing a rigorous testbed to evaluate KD's ability to generalize across different molecular property domains.

**Figure 7:**
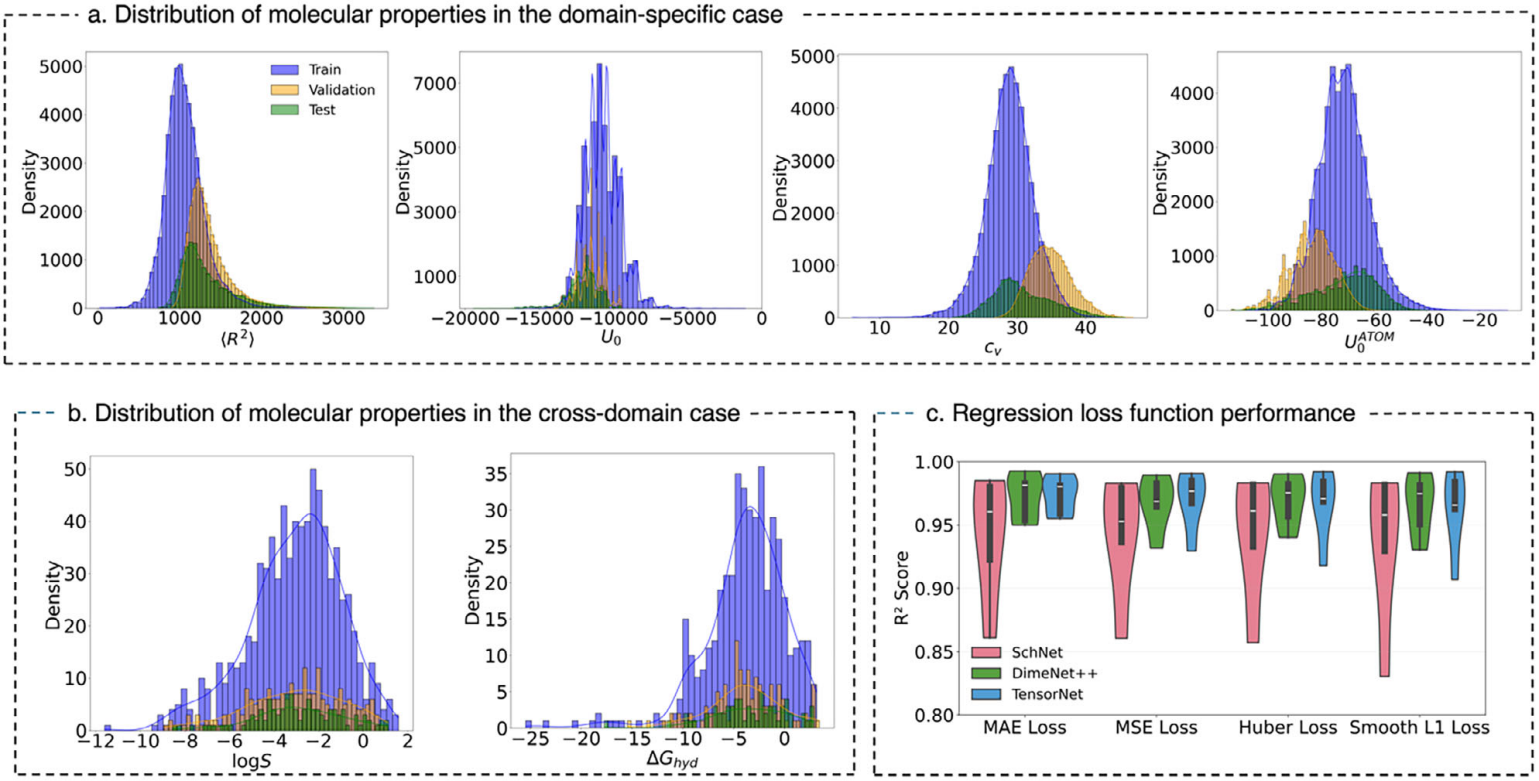
Statistical distributions of molecular properties used in domain‐specific and cross‐domain KD tasks. a) Distributions of selected quantum mechanical properties from the QM9 dataset, including electronic spatial extent (〈*R* ^2^〉), internal energy (*U*_0_), heat capacity (*c_v_*), and atomization energy per atom ($U_{0}^{ATOM}$), across training (blue), validation (orange), and test (green) splits. Note that the excluded properties displayed substantial overlap in their distributions. b) Distributions of experimental properties from the ESOL (log*S*) and FreeSolv (Δ*G_hyd_*) datasets, used in the cross‐domain KD setup. c) Performance of various regression loss functions—MAE Loss, MSE Loss, Huber Loss, and Smooth L1 Loss—across SchNet, DimeNet++, and TensorNet architectures. Each of the violin plots illustrate the distribution of average $R^{2}$ scores across five QM9 properties.

To ensure consistency and reliable evaluation, all datasets were split into training, validation, and test sets using a 70:20:10 ratio, maintaining balanced distributions and comparable evaluation metrics across both domain‐specific and cross‐domain KD tasks, as shown in **Figure** **7**. In the domain‐specific setting with QM9 Figure 7a, the statistical distributions of these selected properties exhibit significant overlap across splits, ensuring representativeness and reducing potential biases in training and evaluation. For cross‐domain KD, Figure 7b presents the distributions of two experimental properties—aqueous solubility (log*S*) from the ESOL dataset and hydration‐free energy (Δ*G_hyd_*) from the FreeSolv dataset. The statistical overlap between training, validation, and test splits ensures representativeness, reducing potential biases in model evaluation. For cross‐domain KD, differences in molecular distributions across QM9, ESOL, and FreeSolv provide a challenging test for KD generalization. This split strategy provides a realistic assessment of KD's generalization capabilities across datasets with varying distributions and noise levels, facilitating meaningful comparisons between domain‐specific and cross‐domain scenarios.

### Training and Evaluation of Knowledge‐Distilled Models

The training and evaluation framework was designed to assess the impact of KD on regression‐based molecular property predictions, focusing on both domain‐specific and cross‐domain tasks. The student and teacher models across all the three architectures aimed to minimize the mean absolute error (MAE) loss shown in Equation (2), which is particularly effective for regression tasks involving molecular properties.^[^ ^22^, ^23^ ^]^ Moreover, MAE loss is preferred for molecular property prediction because it is less sensitive to outliers than other loss functions, such as MSE, ensuring stable optimization, particularly for molecular datasets where numerical values span multiple orders of magnitude.

Additionally, to further validate the effectiveness of MAE loss, comprehensive evaluation was conducted across the five selected QM9 properties used to train the teacher models. As shown in the bottom‐right panel of Figure 7, this analysis confirms that MAE loss consistently outperforms other regression loss functions, such as mean squared error, Huber, and Smooth L1, across all three architectures—SchNet, DimeNet++, and TensorNet. The results reveal that MAE loss yields the highest average $R^{2}$ scores while exhibiting noticeably lower variability, reinforcing its reliability in producing stable predictions. This advantage was particularly pronounced in TensorNet and DimeNet++, where the reduced spread of $R^{2}$ values further emphasize MAE's ability to ensure consistent performance across different molecular properties.

The MAE loss (*L_MAE_*) is defined as:

(2) $$L_{MAE}=\frac{1}{N}\;\sum_{i=1}^{N}\left|y_{i,true}-y_{i,pred}\right|$$

where *y*_*i*,*true*_ are ground truth values for the properties, *y*_*i*,*pred*_ are the corresponding predicted values, and *N* is the number samples in the batch. Note that the teacher models generated both predictions and embeddings that were subsequently transferred to student models.

Student models, in particular, were trained using a combined loss function (*L_total_*). This loss consisted of a regression term, *L_MAE_*, and a distillation term, called the cosine similarity loss (*L_KD_*). Cosine similarity loss was employed to enhance embedding alignment between teacher and student models, ensuring that the distilled representations retained structural consistency while preserving meaningful molecular relationships. This approach was particularly advantageous in regression tasks, where maintaining relative distances in latent space is crucial for accurate property prediction.^[^ ^39^ ^]^

The *L_KD_* loss is defined in Equation (3) as:

(3) $$L_{KD}=1-\cos\left(e_{T},\;e_{S}\right)$$

where *e_T_* and *e_S_* represent the embeddings of the teacher and student models, respectively.

The total uncertainty‐weighed loss function^[^ ^58^ ^]^ (*L_total_*) is expressed below in Equation (4) as:

(4) $$L_{total}=\frac{L_{MAE}}{2\sigma_{1}^{2}}+\frac{L_{KD}}{2\sigma_{2}^{2}}+\log\sigma_{1}+\log\sigma_{2}$$

where σ_1_ and σ_2_ are learnable parameters that dynamically adjust the contribution of each loss term based on task uncertainty. This uncertainty‐based weighting ensures an adaptive balance between regression accuracy and embedding alignment, allowing the model to leverage the benefits of KD without requiring manually tuned weight coefficients. The optimal loss weighting is learned during training rather than being fixed.

The effectiveness of KD was evaluated using the coefficient of determination *R* ^2^, a metric that quantifies the proportion of variance in the ground truth explained by the model's predictions. *R* ^2^ is calculated using Equation (5):

(5) $$R^{2}=1-\frac{\sum_{i=1}^{N}\left(y_{i,true}-y_{i,pred}\right)}{\sum_{i=1}^{N}\left(y_{i,true}-\;\bar{y}\right)}$$

where $\bar{y}$ is the mean of the ground truth values. Higher *R* ^2^ values indicate better alignment between predictions and ground truth, with a maximum value of 1 representing perfect predictions. While primarily performance was analyzed using relative $R^{2}$ deviation (see Equation (1)) to compare improvements across models, absolute $R^{2}$ values for all teacher and student models are also reported in Table S1 (see Supporting Information) to provide a direct assessment of predictive accuracy.

Hyperparameter tuning was performed using Optuna^[^ ^57^ ^]^ to optimize key parameters, including the learning rate and batch size. The Adam optimizer^[^ ^59^ ^]^ with a learning rate of 5 × 10^−5^ and a batch size of 64, determined through the tuning process, was employed for all experiments. Training and evaluation were conducted on NVIDIA A100 GPUs with 40 GB memory, ensuring consistency and efficiency across all tasks.

## Conflict of Interest

The authors declare no conflict of interest.

## Author Contributions

R.S. conducted the model training and analysis and wrote the manuscript. F.Y. conceptualized the study, secured funding, managed the project, and edited the manuscript.

## Supporting information



Supporting Information

## Data Availability

The datasets used in this study, including QM9, ESOL, and FreeSolv, are publicly available through PyTorch Geometric. QM9 can be accessed at https://pytorch‐geometric.readthedocs.io/en/latest/generated/torch_geometric.datasets.QM9.html, while ESOL and FreeSolv are available through the MoleculeNet benchmark at https://pytorch‐geometric.readthedocs.io/en/2.5.0/generated/torch_geometric.datasets.MoleculeNet.html. Further details on dataset preprocessing and usage are provided in the Methods section. Additionally, the full implementation of our knowledge distillation framework, including dataset preprocessing, model training, and evaluation scripts, is publicly available at https://github.com/PEESEgroup/Knowledge‐Distillation‐For‐Molecular‐Properties.
