# Supplementary material for: Knowledge Distillation for Molecular Property Prediction: A Scalability Analysis
Source: Adv Sci (Weinh). 2025 Apr 9;12(22):2503271. doi: 10.1002/advs.202503271 (PMC12165064; doi:10.1002/advs.202503271)
Supplement: Supplementary file 1 — Supporting Information [file ADVS-12-2503271-s001.pdf]

ADVANCED SCIENCE
Open Access

Supporting Information

for *Adv. Sci.*, DOI 10.1002/advs.202503271

Knowledge Distillation for Molecular Property Prediction: A Scalability Analysis

*Rahul Sheshanarayana and Fengqi You**

Supporting Information

Knowledge Distillation for Molecular Property Prediction: A Scalability Analysis

Rahul Sheshanarayana[1], *Fengqi You*[1,2,3,4*]

[1] Systems Engineering, Cornell University, Ithaca, New York 14853, USA

[2] Robert Frederick Smith School of Chemical and Biomolecular Engineering, Cornell University, Ithaca, New York 14853, USA

[3] Cornell University AI for Science Institute, Cornell University, Ithaca New York 14853, USA

[4] Cornell AI for Sustainability Initiative (CAISI), Cornell University, Ithaca New York 14853, USA

Table of Contents

S1. Impact of knowledge distillation on QM9-like vs non-QM9-like molecules in ESOL S2

S2. Distribution of log$S$ values across QM9-like and non-QM9-like molecules.. S3

S3. Raw R^2 values for all teacher and student models S4

[*] Corresponding author E-mail: fengqi.you@cornell.edu

S1. Impact of knowledge distillation on QM9-like vs non-QM9-like molecules in ESOL

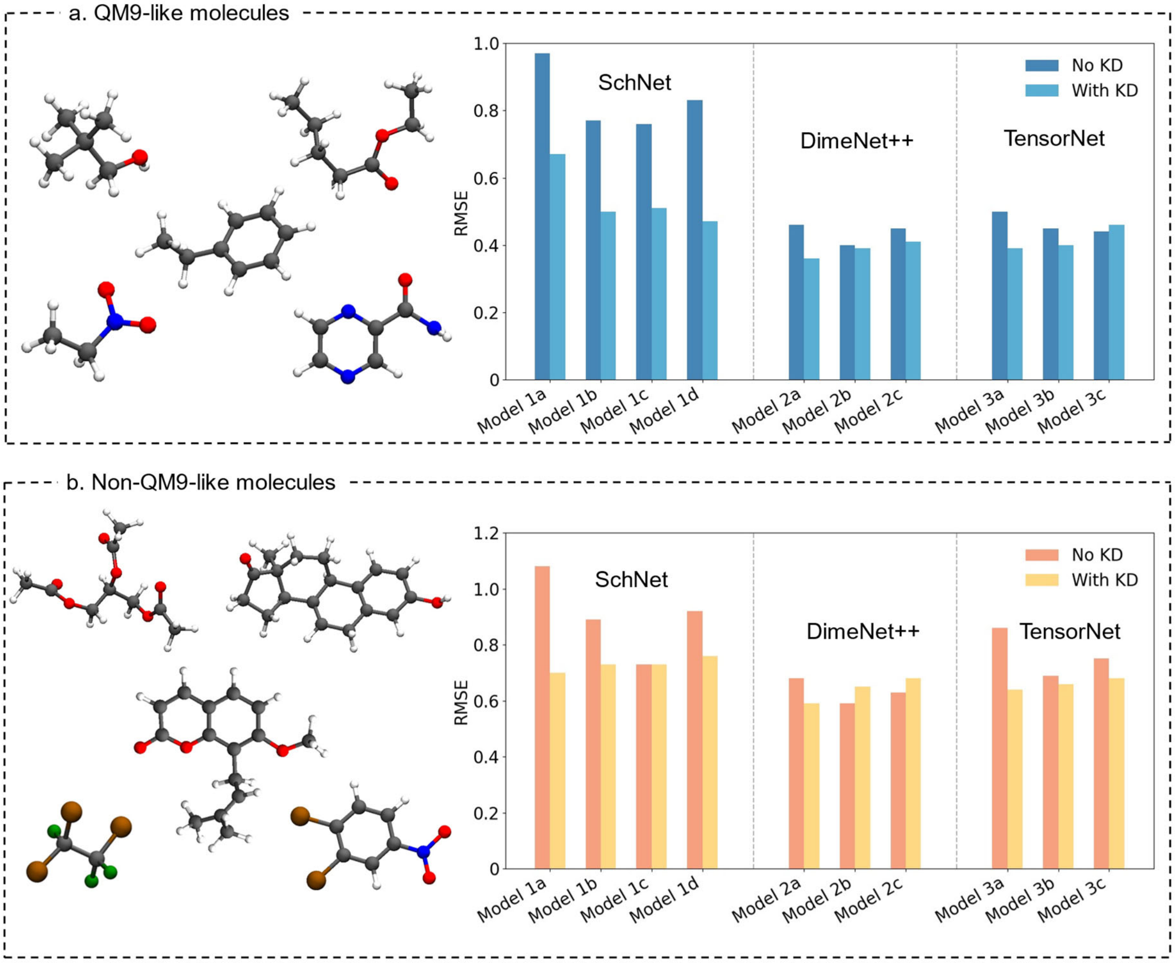

Figure S1: Visualization of QM9-like and non-QM9-like molecules from the ESOL dataset alongside RMSE comparisons of student models trained with and without knowledge distillation (KD). QM9-like molecules (a, left) are structurally similar to QM9 compounds, consisting primarily of carbon (gray), hydrogen (white), oxygen (red), and nitrogen (blue), with up to nine heavy atoms (C, O, N, F). The corresponding RMSE values for SchNet, DimeNet++, and TensorNet models (right) show the effect of KD on QM9-like molecules. Non-QM9-like molecules (b, left) contain additional elements such as fluorine (green), chlorine (brown), sulfur, or phosphorus and often exceed the size constraints of QM9, introducing greater structural diversity. The RMSE values (right) indicate the impact of KD on non-QM9-like molecules. The results demonstrate that KD improves performance across all models, with a more pronounced effect on non-QM9-like molecules.

S2. Distribution of log*S* values across QM9-like and non-QM9-like molecules

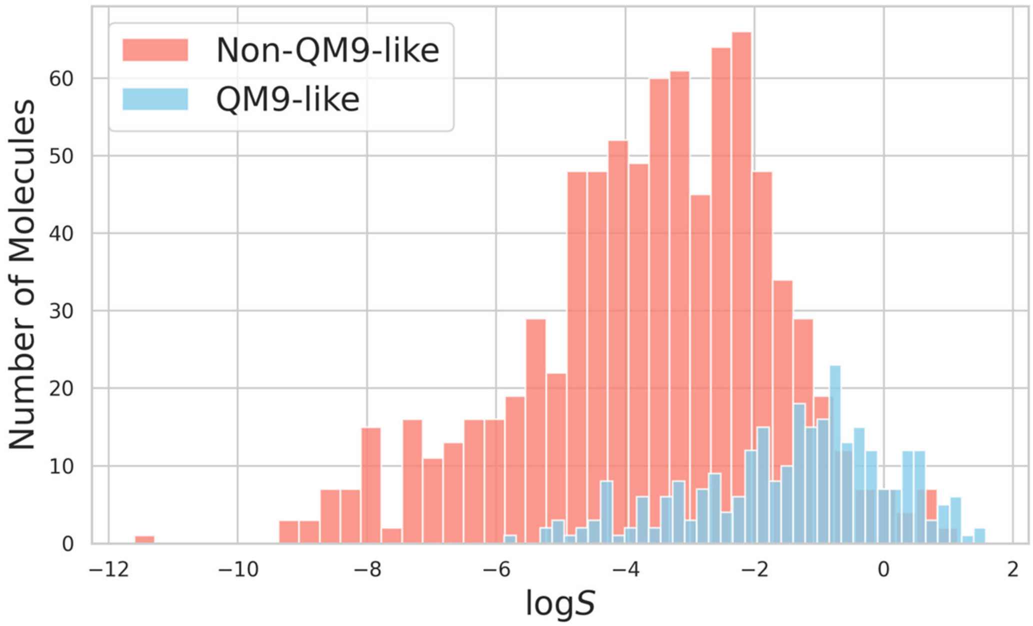

Figure S2: Histogram of log*S* values for QM9-like and non-QM9-like molecules in the ESOL dataset. QM9-like molecules are limited to small organic compounds composed of C, H, O, N, and F and tend to exhibit higher solubility, clustering around a narrower range of log*S* values. In contrast, non-QM9-like molecules—which contain additional elements such as Cl, S, or P and exhibit greater structural diversity—span a broader and more negative log*S* distribution

S3. Raw R^2 values for all teacher and student models

Table S1: Teacher and student model performance across domain-specific (QM9) and cross-domain (ESOL/FreeSolv) datasets. The table reports the R^2 values for QM9, ESOL, and FreeSolv datasets. Note that for QM9, student R^2 values are uniformly averaged over 10 test properties, while teacher R^2 values are averaged over the 5 QM9 properties it was trained on (test set).

Architecture	Model type	Model name	Model Parameters		R^2					
					QM9		ESOL		FreeSolv	
			QM9	ESOL/FreeSolv	Without KD	With KD	Without KD	With KD	Without KD	With KD
SchNet	Teacher	-	456,069		0.94					
	Student	Model 1a	136,714	136,129	0.90	0.92	0.74	0.87	0.89	0.92
		Model 1b	152,650	152,065	0.82	0.88	0.83	0.84	0.84	0.86
		Model 1c	186,826	186,241	0.91	0.88	0.68	0.82	0.82	0.90
		Model 1d	264,394	263,809	0.85	0.90	0.31	0.84	0.90	0.89
DimeNet++	Teacher	-	737,158		0.97					
	Student	Model 2a	237,510	236,934	0.82	0.94	0.83	0.81	0.86	0.93
		Model 2b	404,326	403,462	0.94	0.97	0.82	0.85	0.92	0.91
		Model 2c	571,142	569,990	0.95	0.91	0.77	0.81	0.91	0.94
TensorNet	Teacher	-	1,230,725		0.97					
	Student	Model 3a	528,650	527,489	0.98	0.98	0.84	0.85	0.90	0.92
		Model 3b	762,890	761,729	0.99	0.98	0.84	0.85	0.91	0.94
		Model 3c	997,130	995,969	0.98	0.99	0.76	0.85	0.92	0.91
